# Comments on the review article ‘Antidromic vasodilatation and the migraine mechanism’ by Geppetti et al*.* in the Journal of Headache and Pain 2012; 13:103–111

**DOI:** 10.1007/s10194-012-0431-z

**Published:** 2012-03-18

**Authors:** Elliot Shevel

**Affiliations:** The Headache Clinic, Parktown, South Africa

The authors reviewed the role of antidromic vasodilatation in migraine, and concluded that the weight of evidence shows that the concept of antidromic vasodilatation in migraine cannot be discarded [1]. Unfortunately they included references that they wrongly claimed mitigate against the concept, weakening their position unnecessarily.

In the section entitled ‘Neurogenic vasodilatation in migraine’, they state that Graham and Wolff’s proposal that the headache phase of migraine is associated with dilatation of the superficial temporal artery, was subsequently challenged by failure to measure significant vasodilatation during migraine (a) using Doppler flowmetry [2], and (b) using magnetic resonance angiography [3]. In fact in neither of these studies were the terminal branches of the external carotid artery studied. Zwetsloot studied the only middle cerebral artery and not the extracranial vessels, and Schoonman studied the intracranial vessels, and the last 10 mm of the external carotid *before* it divides into its terminal branches. This section of the external carotid artery has never been implicated in migraine, and is therefore irrelevant in any discussion of Graham and Woolf’s proven theory.

The authors also cite the ‘lack of correlation between the degree of vasodilatation and the severity of headache’ as possibly challenging Woolf’s theory. Both studies that they cite, however, were on headache induced in healthy volunteers [4, 5], and not in migraineurs. Graham and Woolf’s findings relate to the temporal arteries in migraineurs, and should not be compared to the findings in non-migraineurs.

The conclusion of Geppetti et al*.* that the concept of antidromic vasodilatation is valid is strengthened when the incorrect references are removed from the equation.
